# The Effect of a Patient Activation Tailored Intervention on Type 2 Diabetes Self-Management and Clinical Outcomes: A Study from Saudi Arabian Primary Care Settings

**DOI:** 10.1155/2023/2074560

**Published:** 2023-11-27

**Authors:** Nasser Almutairi, Vinod Gopaldasani, Hassan Hosseinzadeh

**Affiliations:** ^1^School of Health & Society, University of Wollongong, NSW, Australia; ^2^Public Health Sector, Ministry of Health, Riyadh, Saudi Arabia

## Abstract

**Background:**

Type 2 diabetes mellitus (T2DM) is a global public health challenge. T2DM self-management, including diet, physical activity, blood glucose self-monitoring, foot care, and adherence to medication, is considered a primary tool for managing diabetes. Patient activation, an individual's knowledge, skill, and confidence in managing their health and healthcare, was recognized to be associated with better T2DM self-management and clinical outcomes. Patient activation intervention has been described as a potential approach for enhancing chronic disease self-management. This study is aimed at examining the effect of a patient activation-tailored intervention on T2DM self-management and clinical outcomes in primary care settings in Saudi Arabia.

**Method:**

A pre- and postintervention study was conducted among ≥18 years old T2DM patients attending primary healthcare centers in Saudi Arabia. Collected data included demographics, clinical data, the Patient Activation Measure (PAM), the Summary of Diabetes Self-Care Activities (SDSCA), the diabetes knowledge test (DKT2), the problem area in diabetes test (PAID-5), and the diabetes quality of life test (DQOL). The intervention was tailored based on the participants' patient activation level. The intervention consisted of monthly face-to-face sessions for three months and a telephone follow-up per month for three months postintervention. Descriptive statistics, a paired sample *t*-test for scale variables, and Wilcoxon's signed-rank test for categorical variables were used for data analysis.

**Results:**

A total of 82 patients, mostly female (61%) with a mean age of 51.3 ± 9.9 years old, completed baseline and postintervention surveys. After six months of intervention, there was a significant change in patient activation score from 54.74 to 61.58 (*p* < 0.001), hemoglobin A1c (HbA1c) from 8.38 to 7.55 (*p* < 0.001), and body mass index (BMI) from 30.90 to 29.16 (*p* < 0.001). Also, there was a significant change in SDSCA scores (diet from 3.12 to 3.67, exercise from 2.54 to 3.49, and blood glucose self-testing from 2.37 to 3.24) (*p* < 0.001) and DKT from 6.29 to 7.22 (*p* = 0.01).

**Conclusion:**

Our findings suggested that tailoring interventions based on patients' activation levels is more likely to yield promising T2DM self-management and clinical outcomes.

## 1. Introduction and Background

Diabetes mellitus (DM) is a significant public health issue. In 2021, it was estimated that 537 million adults were living with diabetes worldwide, and it is projected to increase to 783 million by 2045 [1]. Type 2 diabetes mellitus (T2DM) is the predominant type of diabetes and constitutes 90% of all individuals diagnosed with diabetes [2]. Diabetes risk factors include obesity, a sedentary lifestyle, an unhealthy diet, population growth, aging, and urbanization [3]. In the Middle East and North Africa region, Saudi Arabia has a remarkably high prevalence of diabetes among adults aged 20-79 years at 18.7%, which is nearly double the global prevalence of 10.5%. It is also expected to rise to 21.4% by 2045 [1].

Diabetes self-management is the primary element for controlling and managing diabetes [4]. It refers to the person's ability to manage T2DM-related symptoms and lifestyle changes [5]. This involves several activities such as diet, physical activity, blood glucose self-monitoring, foot care, and adherence to medication [6]. Patient activation is a major driver of self-management. It refers to an individual's knowledge, skill, and confidence in managing their health and healthcare [7]. Literature suggests that people with high levels of activation tend to demonstrate better self-management behaviors, including diet, physical activity, and adherence to a treatment plan [7–12], and optimal clinical outcomes, including glycated hemoglobin A1C (HbA1c), high-density lipoproteins (HDL), blood pressure, and triglycerides [8, 13, 14].

The effectiveness of patient activation intervention on T2DM self-management behaviors and clinical outcomes has been established by several studies [15–17]. For example, Bolen et al. found that patient activation intervention can improve glycemic control, body weight, and systolic blood pressure [16]. Despite the increasing evidence demonstrating the association between patient activation and T2DM self-management behavioral and clinical outcomes, our literature review did not identify any study that has examined the effectiveness of a patient activation intervention on T2DM self-management and clinical outcomes in Saudi Arabia. In addition, most of the conducted interventions in the country are traditional passive educational programs that focus on providing information only instead of building patients' skills and confidence, enabling them to engage in their healthcare plans. For instance, the interventions in certain studies were educational programs focusing mainly on providing information about diabetes, its risk factors and complications, medication, and nutrition [18, 19]. Therefore, this study is an attempt to fill this gap by exploring the effect of a small-scale patient activation intervention on T2DM self-management in primary care settings in Saudi Arabia.

## 2. Methodology

### 2.1. Study Setting and Design

Primary healthcare centers play a significant role in Saudi Arabia's healthcare system, as they serve as the initial touchpoint for individuals seeking medical care. These centers offer a wide range of healthcare services, which are administered by a diverse team of healthcare professionals, including doctors, nurses, pharmacists, dentists, epidemiologists, and administrators. Among these professionals, the doctor assumes a crucial responsibility in delivering primary care services. They can be general practitioners, family medicine specialists, or consultants. This was conducted in primary healthcare centers in Alrass City, Saudi Arabia, from November 2019 to June 2020. The study design was a pre- and postintervention pilot study. The convenience sampling method was utilized to recruit patients attending primary healthcare noncommunicable diseases clinics. Literature suggested that a minimum of 25 participants per group is required for pilot studies [20]. Therefore, in this study, 100 participants were recruited.

### 2.2. Doctors Recruitment and Training

Before data collection, doctors in participating primary healthcare centers were invited to participate in the study. The main researcher has visited the doctors who agreed to participate at the participating centers and briefed them about the project. The participating doctors delivered the intervention on a voluntary basis. They were not compensated monetarily for their involvement in the research. Instead, their participation was motivated by their commitment to advancing medical knowledge and improving healthcare outcomes for the patients they serve.

Five doctors delivered the intervention, on average, 13-14 participants per doctor. All participating doctors had a minimum of 5 years of experience working with people with chronic conditions. The participating doctors included a diabetologist, a public health specialist, a family medicine specialist, and two general practitioner specialists. They received two individualized training sessions. The first session provided knowledge about the main study concepts, such as T2DM, self-management, patient activation, and ethical issues. The second session equipped the participating doctors with the knowledge and skills to deliver a tailored patient activation intervention, which consisted of the T2DM condition and symptoms, medication, diet, physical activity, and stress management. The training sessions were held from 1 December 2019 to 31 January 2020. Each session lasted three to four hours. A written booklet of the intervention was provided to each participating doctor. The progression of the intervention was monitored by on-site visits by the main researcher. There were two visits in the first month, and then one visit per month for three months. Further training was provided during the visits as needed and aimed to address any challenges raised by the doctors, such as patient retention, patient-provider relationships, and documentation burden.

### 2.3. Patient Recruitment and Data Collection

The healthcare system in Saudi Arabia requires individuals with T2DM to regularly visit noncommunicable disease clinics in primary healthcare centers to monitor their health status. Patients were invited to participate in the study by their doctors during their clinic visits. Participating patients completed a self-reported questionnaire in waiting rooms. Participants' data, including demographic and clinical data, Patient Activation Measure, diabetes self-management behaviors, diabetes-related knowledge, diabetes distress, and diabetes-related quality of life, were collected before and after the intervention. To be eligible to participate in this study, participants had to be (a) aged ≥ 18 years old, (b) diagnosed with T2DM, and (c) registered in one of the participating primary healthcare centers. Eligible participants were excluded if they were not fluent in the Arabic language or had cognitive impairment.

This study was reviewed and approved by the Human Research Ethics Committee of the University of Wollongong (number: 2019/337) and the Regional Research Ethics Committee in Saudi Arabia (number: 604577). All individuals who met the eligibility criteria for participation received a participant information sheet (PIS) and a participant consent form (PCF). These documents provided detailed information about the study's objectives and procedures, the voluntary nature of participation, the right to withdraw, the confidentiality of data, the publication of research findings, the storage of data, and the potential benefits of the research.

### 2.4. Intervention

The intervention consisted of monthly face-to-face, 15-20 minute sessions for three months, followed by a telephone call per month for the next three months. The intervention was delivered and followed by the participating doctors. The first session aimed to assess the participant's needs and provide fundamental knowledge based on their level of activation. The following sessions aimed to follow progress, maintain change, and provide extra support if needed. The intervention was developed and tailored based on the participants' patient activation level, which was collected at baseline. For example, if a participant had a lower level of activation, informational and emotional support would be offered to boost their confidence to initiate a new behavior. Emotional support was tailored to the unique emotional needs and challenges reported by each participant. This personalized approach allows to address specific concerns effectively.

The specific objectives of the intervention were to improve diabetes knowledge, problem-solving and goal-setting skills, self-management, and stress management skills among the participants. The participants played an active role in the intervention through shared decision-making. Further details of the intervention for each level are summarized in Table 1. It was expected that after six months of post-intervention, the participants would have improved activation levels, self-management behaviors, clinical outcomes, diabetes knowledge, and diabetes-related distress and quality of life [10, 15, 21].

### 2.5. Study Measurements

Demographic data included age, gender, education, marital status, employment status, and family income status.

Clinical data included HbA1c, blood pressure, lipid profile including cholesterol and triglycerides, BMI (height and weight), type of medication, comorbidities, hospitalization due to T2DM in the last 24 months, the duration of T2DM, and any previous diabetes education. Lab data were collected from participants' health records.

Patient activation was assessed using the Patient Activation Measure (PAM-13), which is a highly valid and reliable instrument for assessing an individual's knowledge, confidence, and skills. The PAM-13 score ranges between 0 and 100, classifying patients into stage 1 (0 to ≤47), stage 2 (47.1 to 55.1), stage 3 (55.2 to 67), and stage 4 (≥67.1). Stage 1 is the least activated, and stage 4 is highly activated [7, 22]. A license to use PAM-13 was obtained from Insignia Health.

Self-management behaviors were measured using the Summary of Diabetes Self-Care Activities (SDSCA) test, which is a self-reported validated multidimensional tool designed to assess adherence to T2D self-management behaviors [23]. For the purpose of this study, 13 items were utilized to measure six domains: diet (four items), physical activity (two items), blood sugar self-testing (two items), adherence to medication (two items), foot care (two items), and smoking (one item). Participants were asked about their self-management activities during the last seven days [23].

Diabetes-related knowledge was evaluated using the revised brief diabetes knowledge test (DKT2), which is a reliable and valid instrument (Cronbach's alpha = 0.77) developed to measure diabetes knowledge. It consists of two parts: 14 items for general knowledge and nine items for insulin use. Each part can be scored independently. Hence, the first part about general knowledge was used for the present study [24].

Diabetes-related distress was measured using the problem area in diabetes (PAID-5), which is a valid and reliable tool (Cronbach's alpha = 0.86) to measure diabetes-related distress. It consists of five items. Each item uses a five-point response option ranging from 0 “not a problem” to 4 “serious problem” [25, 26].

Quality of life was measured using the diabetes quality of life (DQOL) questionnaire, which is a valid and reliable instrument (Cronbach's alpha = 0.85) designed to assess diabetes-specific quality of life [27]. It consists of 15 items evaluating diabetes-related satisfaction, worries, and impacts.

### 2.6. Measurements Translation and Validation

The available Arabic language versions of SDSCA (*α* = 0.76) and DK2 (0.75) were used [28, 29]. PAM-13, PAID-5, and DQOL were translated into Arabic and validated following the World Health Organization (WHO) guidelines for the translation and adaptation of instruments [30]. The English versions were sent to an independent health professional translator whose mother tongue language is Arabic and who is also familiar with the English terminologies used in the instruments. Then, the Arabic versions were reviewed by an expert panel (consisting of two nurses, a family medicine doctor, the original translator, a diabetic educator, and the primary researcher). The inadequate expressions or concepts were resolved. For example, the term “activation” was translated into two Arabic terms, “**تنشيط**” and “**فعالية**”. The expert panel decided to choose “**فعالية**” as it reflects the active role of the individual more than the other term “**تنشيط**”. The revised Arabic versions were back-translated by an independent, native English-speaking translator with no knowledge of the study. The back-translated versions were compared to the original English version by the expert panel, and they were mostly similar. Then, content validity was assessed by the expert panel, and the final versions were prepared for pretesting. The reliability of the measurements was assessed by calculating Cronbach's alpha scores. The scores were as follows: PAM‐13 = 0.85, SDSCA = 0.73, DKT2 = 0.72, PAID‐5 = 0.88, and DQOL = 0.85.

### 2.7. Data Analysis

Data analysis was conducted by using the Statistical Package for Social Sciences (SPSS) version 26. Descriptive statistics were used to describe the frequency, distribution, mean, and standard deviation. Because of the study design that involved paired data (pre- and postintervention measurements within the same participants), the paired sample *t*-test and the Wilcoxon signed-rank test were conducted to assess the difference between baseline and postintervention outcomes. Statistical significance was determined using a *p* value < 0.05.

## 3. Results

### 3.1. Participants' Characteristics

A total of 100 individuals completed the survey and participated in the intervention. However, only 82 participants completed both baseline and postintervention surveys, indicating that 18 participants withdrew from the study during the intervention phase. The reasons for attrition varied and included loss of follow-up, relocating to another city, family issues, loss of interest, or medical issues.

As presented in Table 2, most participants were female (61%) and aged between 41 and 60 years old (67.1%). The mean age was 51.3 ± 9.9 years old. The vast majority of the participants were married (83.8%) and had a secondary or university education (64.2%). In relation to employment, 40.2% of the participants were employed, and 28% were retired. In regard to family income status, 30.8% of the participants were from low-income families and only 16.7% were from high-income families.

### 3.2. Participants' Baseline Clinical Data

Most participants (70.5%) had poor glycemic control, and approximately 57% had normal total cholesterol and triglyceride. Most participants had normal diastolic (92.7%) and systolic (76.8%) blood pressures. Around half of the participants (51.2%) were obese, and 41.7% were overweight. Most participants were diagnosed with T2DM in the past ten years (65.4%) and were on hypoglycemic pills (78.7%). Our analysis also indicated that nine percent of the participants had been hospitalized due to diabetes in the last two years, and more than one-third had at least one chronic condition besides T2DM (36.6%). The most frequent comorbidity conditions were cardiovascular diseases (65.4%) and respiratory diseases (19.2%).

### 3.3. Change in the Study's Outcomes

#### 3.3.1. Patient Activation Scores and Levels

**Figure 1:**
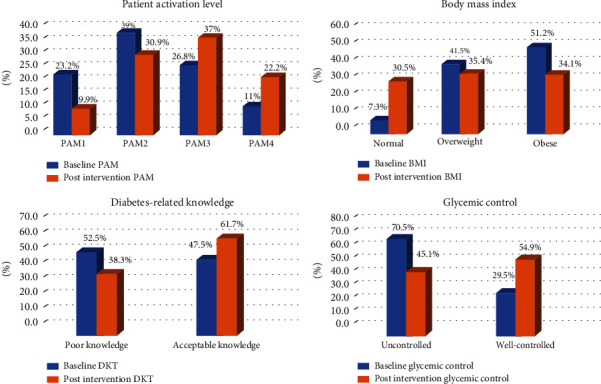
Change in patient activation level, body mass index, diabetes-related knowledge, and glycemic control.

As presented in Table 3, the results showed a significant increase in the PAM score of the participants from a mean of 54.74 (SD = 11.60) at baseline to a mean of 61.58 (SD = 15.69) at six-month follow-up (*t* (80) = −5.30, *p* < 0.001). The mean increase in the PAM scores was 6.83, with a 95% confidence interval ranging from -9.39 to -4.27. As shown in Table 4, there was an overall improvement in PAM level (*z* = −4.44, *p* < 0.001). Forty-two participants (51.8%) increased their PAM level, 30 participants (37.1%) maintained their PAM level, and only 9 participants (11.1%) decreased their PAM level from baseline to six-month postintervention. A comparison between the frequencies of baseline and postintervention PAM levels is illustrated in Figure 1.

#### 3.3.2. Clinical Outcomes

Improvements in clinical outcomes (HbA1c, BMI, diastolic and systolic blood pressures, cholesterol, and triglyceride) are presented in Table 3. The mean HbA1c level decreased from 8.38 (SD = 1.76) at baseline to 7.55 (SD = 1.28) at six-month follow-ups (*t* (66) = 4.76, *p* < 0.001). From a clinical perspective, participants who worsened, maintained, or improved their glycemic control (HbA1c < 7%) were examined by performing the Wilcoxon signed-rank test. As shown in Table 3, twenty-one participants (31.3%) improved their glycemic control, 42 participants (62.7%) maintained their glycemic control, and only 9 participants (13.4%) worsened their glycemic control after six months of follow-ups. A comparison between the frequencies of baseline and postintervention glycemic control is illustrated in Figure 1.

Additionally, there was a significant decrease in BMI from a mean of 30.90 (SD = 5.25) at baseline to a mean of 29.16 (SD = 5.48) at six months of follow-ups (see Table 2). This change of 1.74 was statistically significant (*t* (81) = 6.60, *p* < 0.001) with a 95% confidence interval ranging from 1.21 to 2.25. Thirty-six participants (43.9%) were able to decrease their BMI category, 41 participants (50%) maintained their BMI category, and only 5 participants (6.09%) increased their BMI category after six months of follow-ups (see Table 3). A comparison between the frequencies of baseline and postintervention BMI is illustrated in Figure 1. There was no significant difference in diastolic and systolic blood pressures, total cholesterol, and triglyceride between baseline and postintervention (*p* > 0.05).

#### 3.3.3. Self-Management Behaviors

Table 3 shows the change in the mean score of diabetes self-management behaviors. After the intervention, there was a significant increase in the SDSCA overall score from 3.5 to 4. The mean score for diet increased from 3.12 to 3.67 (0.56), the mean score for exercise increased from 2.54 to 3.49 (0.95), and the blood glucose self-testing mean score increased from 2.37 to 3.24 (0.86) (*p* < 0.001). However, there was no significant change in foot care and adherence to medication (*p* > 0.05).

#### 3.3.4. Diabetes-Related Knowledge Test (DKT)

In terms of DKT, the mean score was 6.29 (SD = 2.83) at baseline and 7.22 (SD = 2.36) at six-month follow-ups (see Table 3). This change of 0.92 was statistically significant (*t* (78) = 2.82, *p* = 0.01). As shown in Table 3, after six months of follow-ups, there were 20 (25.3%) participants who increased their knowledge, 51 (64.5%) maintained their knowledge, and only 8 (10%) participants decreased their knowledge related to diabetes (*z* = −2.27, *p* = 0.02). A comparison between the frequencies of baseline and postintervention DKT is illustrated in Figure 1.

#### 3.3.5. Diabetes-Related Distress Test (PAID-5)

As presented in Table 3, the mean PAID-5 score was 7.43 (SD = 4.82) at baseline and 7.46 (SD = 4.91) at the six-month follow-up. This change of 0.02 was not statistically significant (*p* = 0.96).

#### 3.3.6. Diabetes-Related Quality of Life Test (DQOL)

The mean DQOL score was 31.52 (SD = 8.30) at baseline and 30.05 (SD = 8.28) at the six-month follow-up (see Table 3). This change of 1.47 was not statistically significant (*p* = 0.15).

## 4. Discussion

### 4.1. Change in PAM

This study is aimed at assessing the effectiveness of patient activation-tailored intervention on T2DM self-management behaviors and clinical outcomes. The PAM score improved from 54.74 at baseline to 61.58 at postintervention. An improvement of 6.83 points was both behaviorally and clinically significant. In terms of behavior, it moved the postintervention patient activation level from PAM level 2, where the patient lacks the knowledge and confidence to manage their health, to PAM level 3, where the patient is taking action [10]. In relation to clinical significance, evidence showed that a 1 point increase in the PAM score is associated with a 1.8% increase in good HbA1c control and a 1.7% decrease in the likelihood of hospitalization [31]. Our data also indicated that most of the participants were able to maintain or increase their PAM from low levels to high levels. Similar to previous findings, a greater change was observed among people with lower levels of patient activation (PAM-1 and 2) [32]. This significant finding suggests that low-activated patients should be prioritized in any patient activation interventions in clinical settings.

Previous studies revealed similar results. Hibbard et al. conducted a randomized-controlled trial study on patients with chronic conditions to assess the effectiveness of the patient activation-driven intervention. They found, at six-month follow-up, a significant increase in the PAM score in the intervention group compared to the control group [10]. In another study conducted by Miller et al. in Western Australia, their study revealed that self-management intervention in adults with T2DM significantly improved the PAM score by 9.7 points [33]. Also, another randomized-controlled trial study of people with heart failure in the USA revealed a significant increase in the PAM scores at the six-month follow-up [34].

### 4.2. Change in Clinical Outcomes

Our findings are in line with previous findings in which changes in the PAM resulted in a significant change in HbA1c [14, 31]. There was a significant decrease of 0.83 mg/dL in HbA1c postintervention. This change can be considered clinically significant, as suggested by the literature that each 1% decrease in HbA1c was associated with a 21% decrease in the risk of diabetes-related deaths, a 14% decrease in the risk of myocardial infarction, and a 37% decrease in the risk of microvascular complications [35]. In addition, we found that the vast majority of the participants were able to improve or maintain their glycemic control, and a greater change in HbA1c was observed in people with poor glycemic control. We also found that the proportion of participants with glycemic control nearly doubled at the post-six-month follow-up. These findings are consistent with our previously published systematic literature review of randomized control trials (RCTs) aimed at assessing the effectiveness of patient activation intervention on T2DM glycemic control and self-management behaviors, in which the combined mean decrease in HbA1c was 0.92 and the greater decrease was seen when the mean baseline HbA1c was >10 mg/dL [15].

There was also a significant decrease in BMI following the patient activation intervention. An observed change of 1.47 points moved the postintervention mean BMI score from the obesity category to the overweight category. Interestingly, the proportion of participants with normal weight increased by four times at six-month follow-ups. These results agree with those of Shah et al. findings, which showed that, after six months of home-based patient activation intervention on people with T2DM, BMI significantly decreased by 1.5 kg/m^2^ [36]. Similarly, a meta-analysis of 43 studies assessing the effectiveness of patient activation interventions for adults with T2DM showed that patient activation intervention significantly reduced body weight among 5,749 participants [16].

However, in other clinical outcomes, there was no significant difference in diastolic and systolic blood pressure, total cholesterol, and triglyceride between baseline and postintervention. The results of previous research in this regard are mixed. Mei-Yu et al. conducted a systematic review and meta-analysis aimed at assessing the effects of patient activation interventions on clinical and behavioral outcomes in people with chronic conditions. They found that patient activation interventions significantly improve patients' clinical outcomes, including diastolic and systolic blood pressure, total cholesterol, and triglyceride [21]. However, Bolen et al., in their review, suggested that the improvement was very low [16]. One possible explanation for our results is that the mean baseline readings of diastolic and systolic blood pressure, total cholesterol, and triglyceride were within a normal range, which limits the likelihood of improvement. However, this finding warrants further studies.

### 4.3. Change in Self-Management Behaviors

In line with the literature [15, 21, 37], we found that patient activation intervention improved overall self-management behaviors, particularly diet, physical activity, and blood glucose self-testing. However, adherence to medication and foot care did not significantly improve postintervention. This finding might be due to adherence to medication and foot care baseline scores being the highest among self-management behaviors.

### 4.4. Change in Diabetes-Related Knowledge (DKT)

Consistent with the literature [38], we found that patient activation intervention significantly improved diabetes-related knowledge. The proportion of participants with acceptable diabetes-related knowledge increased from 47.5% at baseline to 61.7% at six-month follow-up. These findings support the validation of PAM in measuring the knowledge required for self-management behavior changes [7].

### 4.5. Change in Diabetes-Related Distress and Diabetes-Related Quality of Life

There was no significant improvement in participants' diabetes-related distress and diabetes-related quality of life following the intervention. However, the current study's findings do not support the previous research. Hibbard et al. and Frosch et al. conducted activation interventions in adults with chronic diseases and reported significant improvement in several outcomes, including health-related quality of life [10, 39]. Another systematic review and meta-analysis study concluded that patient activation intervention significantly improved health-related quality of life in patients with chronic diseases [21]. In addition, with respect to diabetes-related distress, Wallace et al. reported a significant decrease in total diabetes distress and in emotional distress and regimen-related distress subscales [38]. Another real-life observational study was conducted to assess the impacts of person-centered patient activation intervention on people with T2DM. Their findings suggested that, at 1-year follow-up, diabetes distress significantly decreased in 1299 participants [40]. It might be challenging to explain the inconsistency between our findings and previous studies; however, it can be related to the effect of the COVID-19 pandemic during the intervention because the literature suggested that diabetic people are more vulnerable to severe symptoms associated with the COVID-19 virus [41]. Therefore, the COVID-19 pandemic might have increased diabetes-related stress [42–44] and negatively affected the quality of life [45, 46] of the study participants. Further follow-up studies are required.

## 5. Study Limitations and Strengths

This study has some challenges and limitations. Firstly, while our study provides valuable insights, it is crucial to acknowledge that the single-group pilot study design employed in our research may impose constraints on the generalizability of our findings. We primarily focused on a specific patient population within our catchment area, which may not represent the full diversity of the broader population. Additionally, our study was conducted in a primary care setting, so the applicability of our findings to other healthcare contexts may vary. The study also had a limited duration, so interpreting results over longer timeframes should be done cautiously. Future research in diverse settings and populations will be essential to validate and extend our findings, recognizing that the pilot nature of our study has inherent limitations in terms of generalizability. Another limitation was that part of the study was conducted at the time of the COVID-19 pandemic, which limited the movements of the researcher and the participants.

To our knowledge, this research is the first of its kind to examine the effects of patient activation intervention on diabetic patients in Saudi Arabia. The findings of this study may lay the groundwork for further research utilizing various approaches to address other chronic illnesses. Furthermore, the study's participants were thoughtfully selected from multiple primary healthcare centers situated across various neighborhoods, which ensured a wide range of demographic and clinical variables were represented.

## 6. Conclusion

In conclusion, our study highlights the importance of patient activation intervention in improving clinical outcomes and self-management behaviors among individuals with type 2 diabetes. Our findings support previous research that emphasizes the positive results of tailoring interventions based on a patient's activation level. Our tailored patient activation intervention demonstrated significant benefits, including improved glycemic control, increased patient activation, and a trend toward reduced healthcare costs. This indicates that directing resources toward patients based on their activation levels cannot only enhance engagement and satisfaction but also lead to more efficient resource allocation. The implications of our study extend to patients seeking better diabetes management, healthcare providers looking to improve patient care, policymakers shaping healthcare policies, stakeholders investing in healthcare outcomes, and researchers exploring innovative approaches. To successfully implement patient activation interventions in primary care settings, clinicians must have a comprehensive understanding of the benefits and value of using tools like the Patient Activation Measure (PAM). Additionally, clear administration procedures that enhance flexibility and adaptability are essential for effectively integrating patient activation strategies. As we reflect on our research, we recognize the importance of future investigations. Subsequent studies should explore the long-term effects of patient activation interventions, their adaptability to diverse healthcare settings, and their impact on various patient populations. Furthermore, examining the barriers and facilitators to implementation will be instrumental in refining and optimizing similar interventions.

Overall, our study adds to the growing body of evidence supporting patient activation as a crucial factor in improving diabetes management and healthcare outcomes. Our findings can serve as a valuable foundation for future research endeavors, policy development, and healthcare practices aimed at enhancing patient activation and the overall quality of care in primary care settings.

## Figures and Tables

**Table 1:** Patient activation tailored intervention.

	PAM-1	PAM-2	PAM-3	PAM-4
Characteristics	Overwhelmed, disengaged, passive, lack of knowledge, and confidence “My doctor is in charge of my health”	Have some knowledge and low confidence but struggling “I could be doing more”	Good knowledge and confidence Taking action and building self-management skills “I am part of my health care team”	Very good knowledge, confident, adopted and maintained new behavior, but may struggle under stress “I am my own advocate”
Goals	Provide basic knowledge Build confidence through small steps	Increase knowledge Develop self-management skill	Adopt and maintain self-management behaviors	Maintain self-management behaviors and prevent relapse
Activities				
Condition and symptoms	Provide basic information about diabetes, risk factors, red flags, and important diabetes numbers	Build more knowledge about diabetes symptoms Learn self-monitoring skills Increase confidence by taking small step to reduce symptoms	Build and maintain diabetes self-monitoring skills Identify the challenges in managing diabetes Self-reward for healthy change	Maintain diabetes self-management behaviors at or near guideline levels Learn skills to get back on track Troubleshoot the difficult situations in self-management
Medication	Provide basic information about diabetes medication and its side effects Take small step in medication adherence	Build more knowledge about medication and the risk of not being taken as prescribed Take small step to increase medication adherence	Strive for full adherence to medication Provide emotional support to overcome the challenges in medication adherence	Maintain optimal medication adherence Anticipate the difficult situation and learn skills to get back on track
Diet	Provide basic information about healthy diet, carbohydrate, and the link between diet and health Take small step change in diet	Build more knowledge about the recommended diabetes diet Learn weight control skills Take more small step change in diet	Try to follow diet guidelines Learn glycemic index and calorie tools Address problem areas and learn problem-solving skills	Maintain a healthy diet Learn skills to get back on track Set some dietary goals
Physical activity	Provide basic information about physical activity and its benefits Address current physical activity level and take small step to change	Learn skills that help to be active Discuss personal barriers and facilitators of being active Take small step to increase physical activity level	Discuss the elements of a well-rounded fitness plan Try to follow guidelines for physical activity Address the challenges and barriers	Maintain physical activity Anticipate the difficult situation and learn skills to get back on track Set some physical activity goals
Stress management	Provide basic information about what stress is Know stress triggers and levels Take small step to reduce stress	Address personal stress triggers Learn stress coping skills Take small step change in stress management	Full understanding of stress effects Anticipate and avoid stress triggers Master stress coping skills	Follow a healthy lifestyle Track overall health, fitness, emotions, and stress levels Set some stress management goals

Note. The intervention was guided by “Coaching for Activation (CFA), PAM-based guidance to improve health behaviors” provided by Insignia Heath https://flourishcfa.insigniahealth.com/diabetes/.

**Table 2:** Baseline participants' characteristics data (*N* = 82).

Variable	Category	*n*	%
Gender	Male	32	39.0
	Female	50	61.0

Age group	≤40	12	14.6
	41-60	55	67.1
	>60	15	18.3

Marital status^a^	Single	3	3.8
	Married	67	83.8
	Others “divorced and widowed”	10	12.5

Education^a^	No education	16	19.8
	Primary education	13	16.0
	Secondary education	17	21.0
	University education	35	43.2

Employment status	Employed	33	40.2
	Not employed	26	31.7
	Retired	23	28.0

Family income status^b^	Low income	24	30.8
	Middle income	41	52.6
	High income	13	16.7

Glycemic control (HBA1c) (M = 8.38, SD = 1.76) (%)	Controlled (≤7%)	23	29.5
	Uncontrolled (>7%)	55	70.5

Total cholesterol	Normal (<5.18 mmol/L)	44	57.1
	Abnormal (>5.18 mmol/L)	33	42.9

Triglyceride	Normal (≤1.70 mmol/L)	45	57.7
	Abnormal (>1.70 mmol/L)	33	42.3

Diastolic blood pressure	Normal (<90 mmHg)	76	92.7
	Abnormal (≥90 mmHg)	6	7.3

Systolic blood pressure	Normal (<140 mmHg)	63	76.8
	Abnormal (≥140 mmHg)	19	23.2

BMI	Normal weight (18.5-24.9)	6	7.3
	Overweight (25-29.9)	34	41.5
	Obese (≥30)	42	51.2

Diabetes duration	<5 years	29	37.2
	5-10 years	22	28.2
	>10 years	27	34.6

Type of medication	Insulin only	8	9.8
	Oral hypoglycemic agent only	64	78.0
	Insulin + oral hypoglycemic agent	10	12.2

Hospitalization due to DM	Yes	7	9.0
	No	71	91.0

Comorbidities	Yes	26	36.6
	No	45	63.4

Note. BMI = body mass index; M = mean; SD = standard deviation. ^a^Marital status and education were recategorized into new groups for the purpose of this analysis. Age was a continuous data, but it was categorized for the purpose of this analysis. ^b^Family income is per month and classified into three groups: low income (<10000 SAR), middle income (10000-15000 SAR), and high income (>15000 SAR). 1 SAR = 0.35 AUD. Saudi family income data (https://www.stats.gov.sa/sites/default/files/household_income_and_expenditure_survey_2018_en_27-6-2019.pdf).

**Table 3:** Paired sample *t*-test of study's outcomes.

	Baseline (M ± SD)	Postintervention (M ± SD)	Mean difference	*t*	*p*
PAM score	54.75 ± 11.60	61.58 (15.69)	-6.83	-5.30	<0.001
Clinical outcomes					
HbA1c	8.38 ± 1.76	7.55 ± 1.28	0.83	4.76	<0.001
Diastolic blood pressure	75.79 ± 8.09	75.82 ± 7.83	-0.03	-0.03	0.98
Systolic blood pressure	127.71 ± 13.07	124.79 ± 18.73	2.92	1.05	0.55
Cholesterol	4.95 ± 1.15	4.81 ± 1.20	0.15	0.87	0.39
Triglyceride	1.58 ± 0.60	1.62 ± 0.65	-0.04	-0.36	0.72
BMI	30.90 ± 5.25	29.16 ± 5.48	1.74	6.60	<0.001
Self-management behaviors					
General diet	2.89 ± 2.18	3.90 ± 2.16	-1.01	-3.67	<0.001
Specific diet	3.39 ± 1.54	3.46 ± 1.44	-0.07	-0.36	0.72
Overall diet score	3.12 ± 1.57	3.67 ± 1.56	0.56	-2.85	0.006
Exercise	2.54 ± 2.37	3.49 ± 2.52	-0.95	-3.47	<0.001
Blood glucose self-testing	2.37 ± 2.04	3.24 ± 2.43	-0.86	-2.98	<0.001
Foot care	3.30 ± 2.77	3.55 ± 2.69	-0.25	-0.78	0.44
Adherence to medication	6.40 ± 1.76	6.51 ± 1.58	-0.11	-0.59	0.56
Overall score	3.50 ± 1.15	4 ± 1.24	-0.50	-3.70	<0.001
DKT	6.29 ± 2.83	7.22 ± 2.36	-0.92	-2.82	0.01
PAID-5	7.43 ± 4.82	7.46 ± 4.91	-0.02	-0.05	0.96
DQOL	31.52 ± 8.30	30.05 ± 8.28	1.47	1.47	0.15

Note. BMI = body mass index; DKT = diabetes-related knowledge test; PAID-5 = problem area in diabetes; DQOL = diabetes-related quality of life; M = mean; SD = standard deviation.

**Table 4:** Wilcoxon's signed-rank test of change in BMI, glycemic control, PAM level, and DKT.

	Ranks	*N*	Sum of the rank	*z*	*p*
BMI	Negative rank	5	761	-4.81	<0.001
	Positive rank	36	100		
	Ties	41			

Glycemic control	Negative rank	4	13	-3.40	<0.001
	Positive rank	21	13		
	Ties	42			

PAM level	Negative rank	9	25.33	-4.44	<0.001
	Positive rank	42	26.14		
	Ties	30			

DKT	Negative Ranks	8	116	-2.27	0.02
	Positive Ranks	20	29		
	Ties	51			

Note. Negative rank = worsened; positive rank = improved; ties = no change.

## Data Availability

The data used to support the findings of this study are included within the article.
